# High-resolution maps of Swiss apiaries and their applicability to study spatial distribution of bacterial honey bee brood diseases

**DOI:** 10.7717/peerj.6393

**Published:** 2019-01-31

**Authors:** Raphael S. von Büren, Bernadette Oehen, Nikolaus J. Kuhn, Silvio Erler

**Affiliations:** 1 Department of Environmental Sciences, Physical Geography and Environmental Change, Universität Basel, Basel, Switzerland; 2 Research Institute of Organic Agriculture—FiBL, Frick, Switzerland; 3 Institute of Biology, Animal Ecology, Martin-Luther-University Halle-Wittenberg, Halle, Germany

**Keywords:** *Apis mellifera*, Pathogen transmission, Honey bee, Geographic information system, European foulbrood, American foulbrood, Agricultural landscape types, Beekeeping, *Melissococcus plutonius*, *Paenibacillus larvae*

## Abstract

Honey bees directly affect and are influenced by their local environment, in terms of food sources, pollinator densities, pathogen and toxin exposure and climate. Currently, there is a lack of studies analyzing these data with Geographic Information Systems (GIS) to investigate spatial relationships with the environment. Particularly for inter-colonial pathogen transmission, it is known that the likelihood of a healthy colony to become infested (e.g., Varroosis) or infected (e.g., American foulbrood—AFB, European foulbrood—EFB) increases with higher colony density. Whether these transmission paths can actually be asserted at apiary level is largely unknown. Here, we unraveled spatial distribution and high-resolution density of apiaries and bacterial honey bee brood diseases in Switzerland based on available GIS data. Switzerland as ‘model country’ offers the unique opportunity to get apiary data since 2010 owing to compulsory registration for every beekeeper. Further, both destructive bee brood diseases (AFB and EFB) are legally notifiable in Switzerland, and EFB has an epizootic character for the last decades. As governmental data sets have to be ameliorated, raw data from the cantonal agricultural or veterinary offices have been included. We found a mean density of 0.56 apiaries per km^2^, and high resolution spatial analyzes showed strong correlation between density of apiaries and human population density as well as agricultural landscape type. Concerning two bacterial bee brood diseases (AFB, EFB), no significant correlation was detectable with density of apiaries on cantonal level, though a high correlation of EFB cases and apiary density became obvious on higher resolution (district level). Hence, Swiss EFB epizootics seem to have benefited from high apiary densities, promoting the transmission of pathogens by adult bees. The GIS-based method presented here, might also be useful for other bee diseases, anthropogenic or environmental factors affecting bee colonies.

## Introduction

Economically, the Western honey bee (*Apis mellifera*), as well as wild bees (Garibaldi et al., 2013), are the most important insects providing pollination services for human food security and generating valuable bee products for the apicultural sector (Klein et al., 2007; Morse & Calderone, 2000). Bees (*Anthophila*), including honey bee species, are not only indispensable for the agricultural production but also relevant for maintaining and enhancing biodiversity and conservation of wild plants (Ollerton, Winfree & Tarrant, 2011; Peters et al., 2017). Estimated 35% of the global crop production volume, mainly fruits and vegetables, is dependent on insect pollination (Klein et al., 2007). The economic value of the ecosystem service pollination to agriculture has been estimated at 153 billion Euro per year globally, which was 9.5% of the value of the world agricultural production used for human food in 2005 (Gallai et al., 2009).

In spite of this importance for food production, local decline in honey bees is well documented, however globally total colony numbers rise (FAO, 2018; Moritz & Erler, 2016). Several biotic and abiotic factors potentially affect honey bee health (Goulson et al., 2015; Neumann & Carreck, 2010; Potts et al., 2010a), resulting in regional decline of honey bee populations across the United States (Kulhanek et al., 2017; VanEngelsdorp et al., 2009) and Europe (Brodschneider et al., 2018; Moritz & Erler, 2016; Potts et al., 2010b). Multiple causes of colony or apiary losses have been proposed: pathogens (Genersch et al., 2010; Goulson et al., 2015; McMenamin & Genersch, 2015; Stokstad, 2007), pesticides (Gill, Ramos-Rodriguez & Raine, 2012; Goulson et al., 2015; Henry et al., 2012; Sanchez-Bayo et al., 2016), loss of genetic diversity (Meixner et al., 2010), habitat loss and fragmentation (Clermont et al., 2015), political and socio-economic factors (Moritz & Erler, 2016; Smith et al., 2013), climate change (Le Conte & Navajas, 2008), environmental pollution, invasive species (Lautenbach et al., 2012), deficient food resources (Di Pasquale et al., 2016) and combinations of different factors (Alaux et al., 2010).

Honey bees, as well as other eusocial insects, live together as a superorganism in colonies (Seeley, 1989). Having high densities of individuals as well as constant climate and food supply, and the grouping of several bee colonies at the same place (apiary) for a more rational beekeeping, makes honey bees prone to pathogen transmission within (intra-colonial) and between colonies (inter-colonial) (Forfert et al., 2015; Fries & Camazine, 2001; Watanabe, 1987). In particular robbing, drifting and foraging increase the risk of inter-colonial pathogen transmission (Forfert et al., 2015). Other paths for pathogen transmission and persistence are some beekeeping practices like migratory beekeeping (Gordon, Bresolin-Schott & East, 2014; Jara et al., 2018; Simone-Finstrom et al., 2016) but also the exchange of contaminated food/honey and equipment (Fries & Camazine, 2001).

Several studies showed that disease prevalence (e.g., Varroosis, American foulbrood—AFB, European foulbrood—EFB) increases with infected colonies nearby (Belloy et al., 2007; Lindström, Korpela & Fries, 2008; Nolan & Delaplane, 2017), respectively with higher regional colony abundance (Forfert et al., 2016). Whether these transmission paths can actually be asserted at apiary level is still largely unknown. Analyses on the relationship between disease prevalence and density of apiaries have been neglected nearly completely. Only a few studies found evidence for in-field bee pathogen transmission with a high transmission probability between apiaries within at least one kilometer (Belloy et al., 2007; Lindström, Korpela & Fries, 2008; Nolan & Delaplane, 2017).

Comparing all studies describing potential factors that may cause colony losses, it is striking that there is a significant lack of spatial analyses for each of them. Although honey bees are directly influenced by their local environment, only few studies have integrated honey bee data into Geographic Information Systems (GIS) for the purpose of investigating spatial relationships (Camargo et al., 2014; Clermont et al., 2015; Rogers & Staub, 2013; Switanek et al., 2017). However, GIS have great potential to investigate epidemiology of infectious diseases, for instance of bacterial bee brood diseases (Cordoni & Spagnuolo, 2007; Mill et al., 2014; Otten & Otto, 2005; Wilkins, Brown & Cuthbertson, 2007). In medical science and epidemiology, GIS is a powerful and regularly used method (Bithell, 1990; Brooker & Michael, 2000; Davies, Hazelton & Marshall, 2011; Ostfeld, Glass & Keesing, 2005; Pfeiffer & Hugh-Jones, 2002).

For Switzerland, data on colony density and the spatial aspect is known (Fluri, Schenk & Frick, 2004), although with a distribution on very low resolution. Colony densities were described on cantonal level only, with 23-times differences between cantons and an average bee density of 4.7 colonies/km^2^ in 2003 (Fluri, Schenk & Frick, 2004). Unfortunately, this study gave no insight on large inter-cantonal differences, and results should be interpreted with caution as there was no regulated registration of apiaries by law during this time (FSVO, 2010). Consequently, up-to-date data are needed to study spatial distribution of apiaries and their economic importance. Such a high-resolution map illustrating apiary densities is of outstanding relevance to allow for continuative investigations, for example correlational analysis of apiary density and agricultural pesticide use, intensive agriculture (monocultures), disease outbreaks and transmission, or pollination performance (Sutter et al., 2017).

Here, based on available GIS data, we want to unravel spatial distribution and high-resolution density of apiaries in Switzerland on cantonal and municipal level. Further, we want to disentangle whether an increasing density of apiaries actually leads to an increasing probability of disease outbreaks (e.g., AFB, EFB). Switzerland as ‘model country’ offers the unique opportunity to get trustworthy apiary data since 2010 due to compulsory registration for every beekeeper (FSVO, 2010) and sufficient honey bee density compared to other countries (FAO, 2018; Fluri, Schenk & Frick, 2004). Most Swiss beekeepers run their business as hobby with approximately the same number of colonies (according to Fluri, Schenk & Frick (2004) with an average value of 10 colonies/apiary) and migratory beekeeping is operated only by a minority (Federal Office for Agriculture of Switzerland, 2018). Furthermore, both destructive bee brood diseases (AFB and EFB) are legally notifiable bee diseases in Switzerland (data since 1991), and EFB showed an epizootic character for the last decades (FSVO, 2018; Roetschi et al., 2008).

## Materials & Methods

### Apiaries

We obtained geodata (coordinates of apiaries) from the Swiss Federal Office for Agriculture (FOAG). However, among experts, the quality of these FOAG data is doubted. In order to verify the actual quality, we additionally collected raw data for every canton at their agricultural or veterinary offices in the end of 2016. For our main analyses, we created a data set with active apiaries (*‘Switzerland_active’*) containing mainly the cantonal data (not the FOAG data). Since data were not available for some cantons (Appenzell Outer-Rhodes, Appenzell Inner-Rhodes, Basel-City, Basel-District, Jura and Geneva), the cantonal data set was completed by the data of the FOAG resulting in the final *‘Switzerland_active’* data set. Hence, we had three data sets containing active apiaries in Switzerland in 2016:

- *FOAG_active* (from Swiss Federal Office for Agriculture): used for ‘quality-index’
- *Cantonal_active* (from cantonal authorities): used for ‘quality-index’
- *Switzerland_active* (*Cantonal_active* completed by *FOAG_active*): used for main analyses

In the beginning, the geodata were cleaned up from errors. Geodata with incorrect coordinates or duplicates were removed or corrected, if possible. Depending on the canton, 1–5% of all geodata were deleted. The canton of Geneva (GE) had very low quality geodata, leading to the removal of 89% of the data points. For further calculations, inactive apiaries (not actively used by beekeepers) were removed and only the active ones were used. The metadata concerning occupation of apiaries (active/inactive) were nearly always available in the datasets. If not, we assumed that just the active ones were recorded.

The quality of the FOAG data set was quantified in GIS (ArcGIS Desktop 10.3, ESRI Inc.) by comparing the FOAG data set (‘*FOAG_active*’) with the data set of the cantonal authorities (‘*Cantonal_active*’). For this purpose, a ‘quality-index’ was calculated for each canton or municipality based on the ‘swissBOUNDARIES3D’ data set (Swisstopo, 2017). The ‘quality-index’ gives the proportion of the number of apiaries of the FOAG data set, relative to the number of apiaries of the cantonal data set. Thus, a ‘quality-index’ of 100% means that there is the same number of apiaries in both data sets. A ‘quality-index’ >100% indicates more FOAG-apiaries; <100% indicates more apiaries in the cantonal data set.

**Figure 1:**
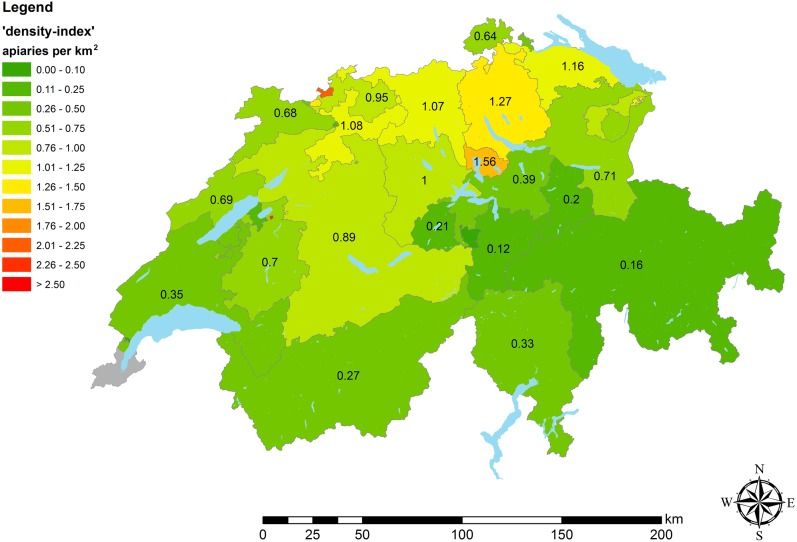
Apiary densities (‘density-indices’) on cantonal level. Different colors illustrate categories of apiary densities (for details see the embedded color code). Low data quality lead to exclusion of apiary data of Geneva (illustrated in grey).******

**Figure 2:**
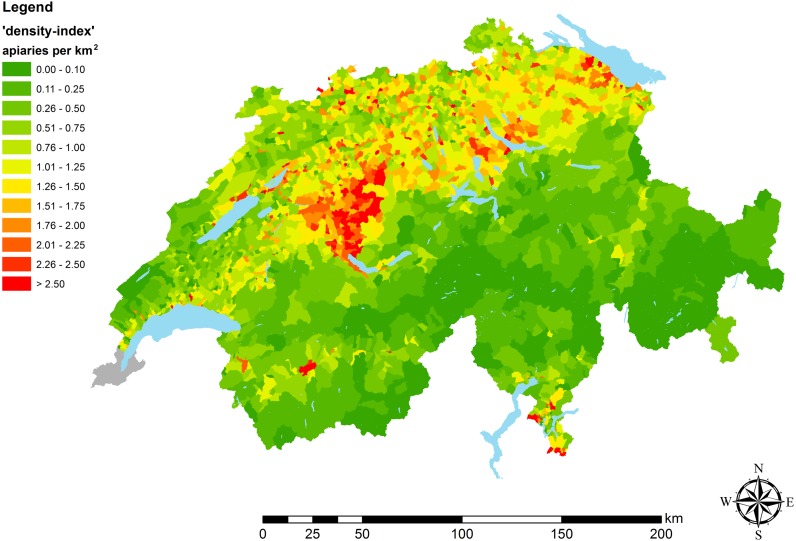
Apiary densities (‘density-indices’) on municipal level. Different colors illustrate categories of apiary densities (for details see the embedded color code). Low data quality lead to exclusion of apiary data of Geneva (illustrated in grey).

**Figure 3:**
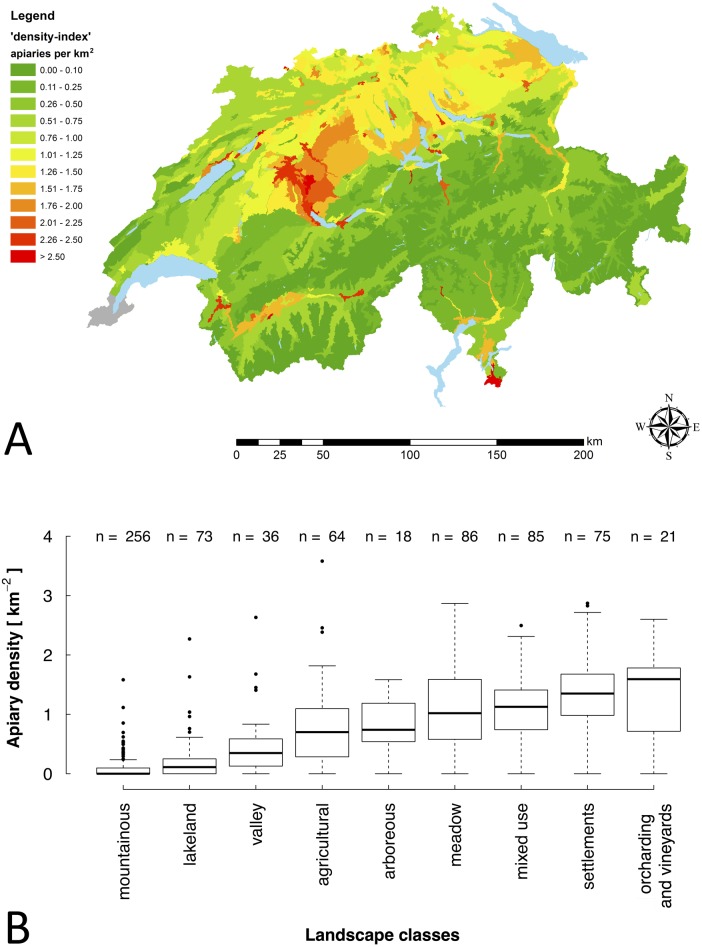
Apiary densities (‘density-indices’) of agricultural landscape types. (A) ‘Density-indices’ for the 55 different types of agricultural landscape defined for Switzerland (Szerencsits et al., 2009). Different colors illustrate categories of apiary densities (for details see the embedded color code). Low data quality lead to exclusion of apiary data of Geneva (illustrated in grey). (B) ‘Density-indices’ for the different agricultural landscape types grouped in nine main landscape classes. Data are shown as box plots with outliers (circles).

Based on the *‘Switzerland_active’* data set, maps were generated in GIS, representing the density of apiaries in 2016. Therefore, at cantonal (average size: 1,588 km^2^), district (average size: 266 km^2^) and municipal (average size: 18 km^2^) level ‘density-indices’ were calculated, giving the number of apiaries per area (km^2^) (Figs. 1 and 2). In addition to these classic geographic area analyses, an agricultural landscape type based analysis was run using the 55 different types of agricultural landscape defined for Switzerland (Federal Office for Agriculture of Switzerland, 2009; Szerencsits et al., 2009) (Fig. 3). To estimate dynamics of apiary densities during the last decade, the current data set at cantonal level was compared to older data (Fluri, Schenk & Frick, 2004). A GIS-map was used to illustrate the percentage change.

In order to investigate possible connections between the distribution of apiaries and occurrences of bee brood diseases (AFB and EFB), the distance to the nearest apiary has to be taken into consideration as well as apiary density. Therefore, GIS-maps were created, which specify the ‘average minimum distance’ to the nearest apiary per study area.

Apiary densities might directly be linked to human settlement or urban areas. To test the hypothesis that higher apiary densities correlate with higher human population densities (higher likelihood for beekeeping activity), a log–log transformed linear model (regression analysis in RStudio 1.0.136 (R 3.4.3); R Core Team, 2018; RStudio Team, 2017) was used, including the calculated apiary ‘density-indices’ and human population density on district level. Human population data came from ‘swissBOUNDARIES3D’ (Swisstopo, 2017) and were processed in ArcGIS.

### Bee brood diseases

The data on honey bee brood diseases in Switzerland were provided by the InfoSM database of the Swiss Federal Food Safety and Veterinary Office—FSVO (FSVO, 2018). The data set used in this study contains all reported epidemiological cases of AFB and EFB since 1991 with information on diagnosis date and place of occurrence (canton, district and municipality). Based on the number of cases for each canton and the number of apiaries per canton, a ‘disease-index’ was calculated using the following mathematical model (mathematical derivation is given in Table S1):

\begin{eqnarray*}\text{` disease-index'} \left[ \text{%} \right] =(1-(1- \frac{#cases}{#months\times #apiaries} )^{#months})\times 100 \end{eqnarray*}

The ‘disease-index’ shows the probability that a particular apiary in the study area was affected at least once during a given period. Testing the hypothesis that bee brood diseases occur more frequently in regions with higher apiary density, a linear model (regression analysis in RStudio) was used including the calculated ‘density-’ and ‘disease-indices’.

However, regression analysis at cantonal level should be interpreted with caution as apiary density within cantons is often distributed very inhomogeneously (Fig. 2). To account for this skewed distribution three cantons were examined at higher resolution (district level). For the following reasons, the cantons of Aargau (AG), Bern (BE) and Solothurn (SO) have been selected as case study objects: (1) very inhomogeneous apiary distribution; (2) extreme values (maxima, minima) for apiary densities in national comparison; (3) high number of reported epidemiological cases and number of apiaries (large sample size); (4) high data quality (‘quality-index’ approximately 100%) and (5) AG, BE, SO are a contiguous study area (exclaves –non-contiguous areas –are not included in the case study).

## Results

### Apiaries

Comparing the FOAG data (‘*FOAG_active*’) with the cantonal data (‘*Cantonal_active*’), to evaluate the quality of the FOAG data, showed that in almost half of all cantons the number of registered apiaries differ only in less than ±5% (Figs. S1 and S2). The other half of the cantons show larger differences, in some cases the ‘quality-index’ is even lower than 50% (e.g., Lucerne: 37%; Zug: 41%; and Neuchâtel: 49%). This locally high discrepancy between FOAG and the cantonal data resulted in the generation of a combined, corrected data set called *‘Switzerland_active’* (for details see ‘Material and Methods’).

This corrected data set used for further analyses, consisting primarily of the cantonal data, comprised in total 23,055 apiaries (for details see Table 1). Based on this data set, an average apiary density (‘density-index’: DI) of 0.56 apiaries per km^2^ was calculated for Switzerland for the year 2016. The highest densities were found in northern central Switzerland (maximum values: Zurich, DI 1.27 and Zug, DI 1.56; Table 1, Fig. 1). The cantons with the lowest ‘density-indices’ are those with high alpine share (minimum values: Uri, DI 0.12 and Grisons, DI 0.16; Table 1, Fig. 1).

**Table 1:** Spatial data (2016) on cantonal level for number of active apiaries, densities and inter-apiaries distances; and bee brood disease announcements (AFB and EFB) in Switzerland (1991–2016). Low data quality lead to exclusion of apiary data of Geneva, indicated by asterisk.

Canton	Active apiaries 2016	‘Density-index’ (km^−2^ )	‘Average minimal distance’ (m)	EFB 1991–2016	EFB ‘disease-index’	AFB 1991–2016	AFB ‘disease-index’
Aargau	1,502	1.07	459	227	14.0%	83	5.4%
Appenzell Outer-Rhodes	201	0.83	514	31	14.3%	1	0.5%
Appenzell Inner-Rhodes	97	0.51	545	46	37.8%	1	1.0%
Basel-District	485	0.95	479	80	15.2%	17	3.4%
Basel-City	38	1.03	418	0	0.0%	4	10.0%
Bern	5,327	0.89	406	3,104	44.2%	398	7.2%
Fribourg	1,123	0.70	484	108	9.2%	148	12.3%
Geneva	*	*	*	14	*	32	*
Glarus	136	0.20	623	72	41.1%	1	0.7%
Grisons	1,125	0.16	594	422	31.3%	411	30.6%
Jura	567	0.68	480	2	0.4%	44	7.5%
Lucerne	1,488	1.00	454	574	32.0%	206	12.9%
Neuchâtel	551	0.69	450	1	0.2%	45	7.8%
Nidwalden	101	0.37	513	8	7.6%	2	2.0%
Obwalden	92	0.21	655	58	46.8%	38	33.9%
St. Gallen	1,447	0.71	479	427	25.6%	33	2.3%
Schaffhausen	192	0.64	600	5	2.6%	10	5.1%
Schwyz	350	0.39	535	36	9.8%	14	3.9%
Solothurn	883	1.08	417	400	36.4%	52	5.7%
Ticino	914	0.33	494	15	1.6%	116	11.9%
Thurgau	1,151	1.16	435	495	35.0%	47	4.0%
Uri	126	0.12	555	32	22.4%	20	14.7%
Vaud	1,113	0.35	605	41	3.6%	221	18.0%
Valais	1,433	0.27	396	283	17.9%	250	16.0%
Zug	372	1.56	385	36	9.2%	35	9.0%
Zurich	2,191	1.27	420	704	27.5%	50	2.3%
**Switzerland**	**23,055**	**0.56**	**460**	**7,221**	**26.91%**	**2,279**	**9.42%**

In contrast, on the high-resolution map on municipal level (Fig. 2), areas with occasionally very high apiary density became visible, which were, due to the low resolution, not displayed on the map at cantonal level (Fig. 1). For instance, in the center of the canton of Bern, an area with consistently high apiary density can be found. There, the municipalities have a density of more than two apiaries per km^2^ (Fig. 2). In a general comparison, it became obvious that in the Swiss midlands and in the pre-Alpine regions, the density of apiaries is higher on average, than in the Alps and the Jura (Table 1; Figs. 1 and 2, S3) (for details according to spatial classification of Swiss landscapes see Szerencsits, 2013). These differences that are related to different regions and human usage were visible, with the highest resolution, on the map including Swiss agricultural landscape types (Fig. 3A). Highest apiary densities are in regions used for fruit and vine cultivation, settlements (e.g., Bern), mixed use and fodder cultivation; whereas lowest densities can be seen for mountainous areas and lakelands (Table 2, Fig. 3B). These differences are highly significant (Kruskal-Wallis ANOVA: *H* = 410.78, *df* = 8, *p* < 0.0001) comparing apiary ‘density-indices’ between nine landscape classes, summarizing the 55 different types of agricultural landscape defined for Switzerland (Table 2, Fig. 3B).

**Table 2:** Densities of apiaries in Switzerland for nine landscape classes summarizing the 55 different types of agricultural landscape defined in Szerencsits et al. (2009).

Landscape class	‘Density-index’ (km^−2^) (median ± SD)
Orcharding and vineyards	1.59 ± 0.72
Settlements	1.35 ± 0.72
Mixed use	1.13 ± 0.53
Meadow	1.02 ± 0.65
Arboreous	0.74 ± 0.48
Agricultural	0.70 ± 0.65
Valley	0.35 ± 0.54
Lakeland	0.11 ± 0.39
Mountainous	0.00 ± 0.18

The comparison of the current density map with the historic data of Fluri, Schenk & Frick (2004) resulted in very heterogeneous percentage changes, varying between −70% (Basel-City) and +103% (Ticino) (Fig. S4). At the moment, putative reasons explaining this high variance are highly speculative and will not be included in the current study. The most obvious factor explaining this observation, is the difference in apiary registration, being compulsory nowadays.

The ‘average minimum distance’ to the nearest apiary is 459.6 m. On cantonal level it varies between 385 m (Zug) and 655 m (Obwalden) (Table 1, Fig. S5). In principle, cantons with a higher ‘density-index’ tend to have a smaller ‘average minimum distance’ (linear regression; *R*^2^ = 0.51, *p* < 0.0001). However, this is not always the case as apiaries are not distributed homogeneously. In mountain regions, for instance, apiaries are often arranged linearly along the agricultural and populated valleys (Fig. 3A, S6, S7). This is evident in the canton of Valais: the ‘average minimum distance’ is very low (396 m), although the ‘density-index’ is not high (DI 0.27) (Table 1).

Comparing apiary density on high resolution level (district) and the human population density, a highly significant correlation was detectable (linear regression; *R*^2^ = 0.64, *p* < 0.0001). The higher the human population density, the more likely higher numbers of beekeepers can be seen, resulting in higher apiary densities (Fig. S8).

### Bee brood diseases

In the period between 1991 and 2016, 9500 cases of bee brood diseases were registered in Switzerland. 81% (7221) of all cases were EFB, 19% (2279) were AFB (Table 1). The temporal analysis of the data confirms previous statements by Roetschi et al. (2008) that EFB has been growing strongly in Switzerland since 1999. It peaked in 2009/10 with 796 cases in 2009 and 987 cases in 2010 respectively. Since 2010, the number of EFB cases is decreasing down to 347 (2015) and 371 (2016) (Figs. S9, S10).

AFB is still declining since 2003 (Fig. S11). Between 1991 and 2003, there were 1403 cases of AFB in Switzerland (62% of all outbreaks), and from 2004 to 2016 there were 876 AFB cases (38% of all outbreaks). While in 1991, 75% of all brood disease cases fell to AFB (65 cases) and only 25% to EFB (21 cases), the increase in EFB cases showed that from 2010 until now, only about 10% of all cases were AFB and about 90% EFB (Fig. S9).

Spatial investigations revealed that in the case of AFB no canton stands out by a higher number of cases than other cantons (Table 1, Fig. S11 ). In stark contrast, a canton stands out clearly in the case of EFB: the canton of Bern with 3104 EFB cases between 1991 and 2016, 43% of all EFB cases in Switzerland (Table 1, Fig. S10). However, the EFB epizootic in Switzerland around the year 2010 was not just a phenomenon in this canton. In most other cantons there was also a high incidence of EFB during this time. Consequently, the disease data (AFB and EFB) of all cantons can be used as a resilient model input to investigate possible interactions between apiary density and disease occurrence. Table 1 and Table S2 show the variables to be analyzed: the density of apiaries and ‘disease-indices’ for AFB and EFB at cantonal (Table 1) and district (Table S2) level.

On cantonal level, there is hardly any evidence for a correlation between apiary density (‘density-indices’) and occurrence of both bee brood diseases (‘disease-indices’) (linear regression; *R*^2^ < 0.001, *p* > 0.9, Table 3). Since apiaries within cantons are distributed very heterogeneously, statistical analyses at cantonal level have to be interpreted with caution. Therefore, further analyses were conducted on district level for three cantons. In the case of AFB, a positive but not significant interaction could be observed (linear regression; *R*^2^ = 0.08, *p* = 0.14, Table 3). Contrary, in the case of EFB, a highly significant correlation (linear regression; *R*^2^ = 0.31, *p* = 0.002, Table 3) between apiary density and occurrence of EFB was detectable. For the three cantons tested on district level (AG, *R*^2^ = 0.54; *p* = 0.009; BE, *R*^2^ = 0.69, *p* = 0.003 and SO, *R*^2^ = 0.41, *p* = 0.09), the pattern of higher apiary densities (‘density-indices’) correlating with higher EFB occurrence (‘disease-indices’) was nearly the same (Table 3).

**Table 3:** Summary of the impact of apiary densities on disease occurrence at cantonal and district level (CH, Switzerland; AG, Aargau; BE, Bern; SO, Solothurn).

Disease	Spatial level	Linear regression			
		intercept	slope	*R*^2^	*p*
AFB	Cantons (CH)	0.11	−0.006	0.0004	0.92
	Districts (AG, BE, SO)	0.02	0.03	0.08	0.14
EFB	Cantons (CH)	0.20	−0.002	0.00002	0.98
	Districts (AG, BE, SO)	−0.001	0.29	0.31	0.002

## Discussion

In order to analyze the key question of this study, namely to investigate a possible correlation between density of apiaries and occurrence of bacterial bee brood diseases, high quality data sets are of great importance. In Switzerland, the Federal Food Safety and Veterinary Office (FSVO) and the Federal Office for Agriculture (FOAG) administer two unique data sets on apiary locations and bee health. Since 2010, beekeepers report the exact location of their apiaries (coordinates) to their cantonal administration (FSVO, 2010). The cantons pass this information on to the FOAG. In addition, it is mandatory for all beekeepers to inform the FSVO on bee diseases: in particular AFB and EFB, as well as the small hive beetle (TSV, 2018). This is the first study in which both data sets are used together.

### Apiaries

The distribution of apiaries at municipal level is very heterogeneous (Fig. 2). At first sight, Alpine cantons (e.g., Grisons and Uri) have a very low apiary density. This is in line with Crane (1992) who found that the upper limit of permanent colonies in the Alps is at an altitude of about 1500 m above sea level. Fluri, Schenk & Frick (2004) and Fluri & Frick (2005) assumed that the low apiary density is linked with the generally lower human population density in these areas and thus a lower number of potential beekeepers. Here, we found as well a very high correlation (Fig. S8) and the lowest apiary density in mountainous areas (Table 2, Figs. 3A, Fig. 3B), supporting this assumption. The spatial analysis in our study revealed that in the Alpine area of Switzerland, apiaries are arranged linearly along the agricultural and populated valleys. In addition, the limited number of suitable sites for apiaries (steep terrain, late sun exposure in spring, avalanche risk) and reduced economic advantages (limited honey production, starvation risk during summer) might be additional reasons for the lower number of beekeepers.

The high apiary densities near midland lakes (e.g., Thurgau, Zug and Zurich) might be explained by the high human population density close to these lakes, but also by the topographically favorable conditions for apiaries and the abundance of foraging sources for bees (Klein et al., 2007), e.g., fruit trees (Lake Constance), vegetable production (Moos near Lake Biel) and vineyards (Lake Biel, Lake Geneva). Even that grapes (*Vitis vinifera*) are self-pollinated and do not depend on honey bees (Kassemeyer & Staudt, 1981), bee colonies are kept in the vicinity of vineyards, as honey bees potentially help to control the grape moth (*Eupoecilia ambiguella, Lobesia botrana*) and pollinate fruit trees, which are often planted at the edges of vineyards (Tautz & Rostas, 2008). Both assumptions could be confirmed by the highest apiary densities in areas with settlement, fruit and wine cultivation (Table 2, Figs. 3A, 3B).

Within the scope of this study, the apiary density was calculated based on the number of apiaries divided by the surface of Switzerland, the canton, the district and the municipality, respectively. Fluri, Schenk & Frick (2004) also calculated honey bee densities. They used the number of colonies, the total surface of Switzerland (41,285 km^2^) and the cantons, and found 4.7 colonies per km^2^ in 2003, with mountains and lakes included. Although the numbers from their study (colony density) are not directly comparable to our study (apiary density), the apiary density in 2003 can be estimated by assuming that each beekeeper owns one apiary with approximately 10 colonies (Fluri, Schenk & Frick, 2004). This means in 2003 Switzerland had an average apiary density of 0.47 per km^2^, being slightly below the density calculated here (0.56 apiaries per km^2^).

According to Fluri, Schenk & Frick (2004) and the FAO (2018), the number of Swiss colonies has been declining since World War II. This development should also be observed in the number of apiaries. However, comparing the data from Fluri, Schenk & Frick (2004) with the cantonal data of 2016 of this study, not such a trend became obvious (Fig. S4). Most of the cantons have a much higher density of apiaries in the more recent data, though these unexpected results call into question the quality of the data, as indicated by Moritz & Erler (2016) for comparable data sets. However, we have to emphasize that in 2003 it was not yet mandatory to register apiaries. Therefore, it can be assumed that not all apiaries were recorded at this time.

### Bee brood diseases

AFB and EFB, both caused by Gram-positive bacteria (AFB: *Paenibacillus larvae*; EFB: *Melissococcus plutonius*), are widely distributed in Switzerland (Forsgren et al., 2018; Gillard, Charriere & Belloy, 2008; Roetschi et al., 2008) and both diseases are potentially lethal to the brood of infected colonies (Forsgren, 2010; Forsgren et al., 2018). Adult bees can act as carriers and spread (in combination with the beekeeper) the disease between colonies and apiaries (Belloy et al., 2007; Lindström, Korpela & Fries, 2008).

Belloy et al. (2007) showed in a field study that almost 40% of bee colonies, between 500 and 1,000 m from a bee colony with EFB symptoms, were infected with *M. plutonius*. This is about the same distance which was found in this study (using data for all of Switzerland) as average minimum distances between apiaries in Switzerland (350–650 m) (Table 1). With the assumption that within apiaries colony distances are usually much lower than between apiaries, the data of both studies showed that pathogen transmission can be highly successful between apiaries and will be even more successful between colonies of each apiary. Belloy et al. (2007) and Forsgren et al. (2005) pointed out that EFB epizootics in Switzerland might have benefited from the high density of bee colonies, which promotes the transmission of the pathogens by adult bees. Further, Forsgren et al. (2005) supposed that the high density leads to nutrition competition between colonies and increases the risk for EFB. According to Belloy et al. (2007), an increased density leads to shorter distances between apiaries, which means that *M. plutonius* can spread easier. Hence, the spatial distribution of apiaries seems to support pathogen transmission by bees and might explain partly the EFB epizootics in Switzerland. Although there is hardly any statistical correlation between EFB outbreak and apiary density at cantonal level, the case study at district level shows a highly significant, positive correlation between the density of apiaries and the probability of disease outbreaks (Table 3).

However, if there are no, or hardly any, infectious *M. plutonius* bacteria in a region, even a high apiary density does not lead to an increased probability of disease outbreaks. This can be seen in the cantons of Basel-City (0 EFB cases), Jura (2), Neuchâtel (1), Schaffhausen (5) and Ticino (15), and might explain why there was no correlation between apiary density and disease probability in the analysis at cantonal level. The absence of a clear effect at cantonal level might also be explained by the fact that some honey bee colonies may be disease resistant (Spivak & Reuter, 2001). In this case, the disease would be spatial enzootic (as known from other European countries, (Forsgren et al., 2018)) whereas in other regions it remains epizootic. Furthermore, the pure presence of the causative agent does not automatically cause a disease outbreak (Belloy et al., 2007; Forsgren et al., 2005). To test the hypothesis that an increased density may not necessarily lead to an increased probability of infection, but to a faster spread of a regional epizootic, all cases of colony infection would have to be provided with precise coordinates (Datta et al., 2013).

This study shows that the assumption of an increased probability of disease outbreaks for bacterial bee brood diseases in regions with high apiary density seems not to apply in the case of AFB. There is no significant correlation at either district or cantonal level (Table 3). It should be noted that in the case of AFB, sample size is relatively small, leading to lower statistical power and the result must be interpreted with caution. Another explanation for the differences between EFB and AFB might be differences in handling the two bee brood diseases. In the case of AFB, a restricted area is defined, which normally covers an area within a radius of 2 km from the contaminated status. All apiaries within the restricted area are checked and any import and export of bees and honeycombs is prohibited for at least 30 days. In the case of EFB, the radius is only 1 km, covering an area four times smaller than the restricted area in the event of an AFB case (TSV, 2018). Taking into account the foraging range of honey bees, the restriction circle of 1 km around an EFB infected apiary might be insufficient to prevent pathogen transmission. *A. mellifera* has a normal foraging range of up to 1.5 km (Beekman et al., 2004; Danner et al., 2016; Danner et al., 2017; Visscher & Seeley, 1982). Lindström, Korpela & Fries (2008) could show that AFB is transmitted almost exclusively within 2 km. The restriction circle of 2 km around an AFB-affected apiary should therefore be sufficiently large to limit the spread of the bee brood disease under normal conditions, even though a complete prevention cannot be guaranteed. In future studies, restriction circles for bacterial bee brood disease outbreaks have to be compared for different European countries and their effectiveness to prevent pathogen transmission. Restriction circles even larger than 2 km might be a future solution. However, with the high apiary density of Switzerland, economic consequences should be considered carefully.

## Conclusions

Switzerland as ‘model country’ offers the unique opportunity for getting trustworthy apiary data since 2010. However, investigating the quality of the FOAG data set has shown that only half of all cantons have high quality data (Fig. S1). Possible reasons might be the following: (1) FOAG data are not up to date, as the cantons seldom forward data on apiaries; (2) a potential loss of information and data may occur when different data formats in different GIS systems are combined; and (3) inactive apiaries may be partially registered as active apiaries, which causes loss of meta information. Although the data set of Switzerland is probably unique in this form, it is still of great importance to fine tune data collection on Swiss apiaries. In addition, today, GIS data on apiaries are available, but the additional information on the number of bee colonies per apiary, which is collected annually, is often not. The density maps created here can serve as a basis for this purpose.

In further studies concerning spatial distribution of bee brood diseases, more cantons have to be evaluated at district level in order to strengthen the power of the results. Further, it would be beneficial if the InfoSM database of the FSVO (FSVO, 2018) start to record coordinates of the epidemiological cases to have GIS information for further investigations on the occurrences of AFB and EFB.

The GIS-based method presented here, can also be applied to other relevant bee diseases (e.g., viruses, *Nosema*, Varroosis), though for most of them infection data on apiary level will be available less likely. Ministries, agricultural research institutes and others might use the method to test for coherences of apiary densities and the use of pesticides, eutrophication, area of monoculture, etc., to discover regional impact of each factor.

##  Supplemental Information

10.7717/peerj.6393/supp-1 Supplemental Information 1 Supplementary Table and Figures

10.7717/peerj.6393/supp-2 Supplemental Information 2 Disease cases with date of diagnosis, disease, canton, district and municipality

## Additional Information and Declarations

### Data Availability

The following information was supplied regarding data availability: The raw data are available in a Supplemental File.
