# Supplementary material for: High-resolution maps of Swiss apiaries and their applicability to study spatial distribution of bacterial honey bee brood diseases
Source: PeerJ. 2019 Jan 31;7:e6393. doi: 10.7717/peerj.6393 (PMC6360077; doi:10.7717/peerj.6393)
Supplement: Supplemental Information 1 [file peerj-07-6393-s001.pdf]

Supplementary Material

Table S1: Mathematical derivation of the formula for the 'disease-index'.

Steps of mathematical derivation

1)	**Problem:**<br>We want to calculate an index which indicates the probability that a particular apiary in the study area was affected at least once (by AFB or EFB) during the last 26 years (1991-2016). Given are the number of disease outbreaks (in the last 26 years) as well as the number of apiaries (end of 2016).
2)	**Annotations:**<br>n: number of apiaries<br>k: number of disease outbreaks in the last 26 years (312 months)
3)	**Mathematical derivation of the formula:**<br>The model assumption is that all data are evenly distributed. Furthermore, a certain apiary should have exactly once a month the opportunity to have a disease outbreak. It either stays healthy for the whole month or shows symptoms, which is reported as one disease outbreak.<br>Due to the evenly distributed data required in the model, there are $\frac{k}{312}$ disease outbreaks per month. The probability that one out of n apiaries will show a disease outbreak in a given month is $\frac{1}{n} \times \frac{k}{312} = \frac{k}{312n}$. The probability q that the same apiary stays healthy in a given month is $q = 1 - \frac{k}{312n}$. The probability that it remains healthy for 26 years (312 month) is thus $q^{312} = (1 - \frac{k}{312n})^{312}$. The probability p that it has a disease outbreak at least once during the entire period is: $p = 1 - q^{312} = 1 - (1 - \frac{k}{312n})^{312}$. Multiplied by the factor 100, the probability ('disease-index') is given in %.
4)	**Example:**<br>Probability that a particular apiary in the canton of Bern was affected at least once (by EFB) during the last 26 years (1991-2016) → 'disease-index' for EFB in the canton of Bern (1991-2016)<br><br>*n: number of apiaries* *5327 (Table 1)*<br>*k: number of EFB outbreaks in the last 26 years (312 month)* *3104 (Table 1)*<br><br>**'disease-index'** = $(1 - \left(1 - \frac{k}{312n}\right)^{312}) \times 100$ = $\left(1 - \left(1 - \frac{3104}{312 \times 5327}\right)^{312}\right) \times 100 = \mathbf{44.2}$ %

Table S2: Spatial data on district level for number of active apiaries and densities; and bee brood diseases announcements (AFB and EFB) in the contiguous study area (without exclaves) Bern (BE), Solothurn (SO) and Aargau (AG) (1991-2016).

		Active apiaries 2016	'Density-index' [km^{-2}]	EFB 1991-2016	EFB 'disease-index'	AFB 1991-2016	AFB 'disease-index'
**Berner Jura**	**BE**	303	0.56	14	4.5%	7	2.3%
**Bern-Mittelland**	**BE**	1,577	1.67	1,057	48.9%	108	6.6%
**Biel/Bienne**	**BE**	149	1.55	120	55.4%	23	14.3%
**Emmental**	**BE**	1,142	1.65	840	52.1%	20	1.7%
**Frutigen-Niedersimmental**	**BE**	292	0.38	78	23.5%	20	6.6%
**Interlaken-Oberhasli**	**BE**	301	0.25	119	32.7%	19	6.1%
**Oberaargau**	**BE**	534	1.61	307	43.8%	9	1.7%
**Obersimmental-Saanen**	**BE**	188	0.33	28	13.8%	11	5.7%
**Seeland**	**BE**	355	1.06	129	30.5%	18	4.9%
**Thun**	**BE**	479	1.49	412	57.7%	15	3.1%
**Balsthal-Thal**	**SO**	84	0.60	41	38.6%	0	0.0%
**Bucheggberg**	**SO**	79	1.26	50	46.9%	3	3.7%
**Gäu (and Balsthal-Gäu)**	**SO**	58	0.94	40	49.9%	0	0.0%
**Gösgen**	**SO**	80	1.17	18	20.2%	2	2.5%
**Lebern**	**SO**	136	1.16	138	63.8%	22	14.9%
**Olten**	**SO**	72	0.89	30	34.1%	0	0.0%
**Solothurn**	**SO**	13	2.07	19	76.9%	3	20.6%
**Wasseramt**	**SO**	80	1.05	64	55.1%	6	7.2%
**Aarau**	**AG**	127	1.22	18	13.2%	5	3.9%
**Baden**	**AG**	147	0.96	4	2.7%	6	4.0%
**Bremgarten**	**AG**	103	0.88	4	3.8%	13	11.9%
**Brugg**	**AG**	148	1.02	1	0.7%	15	9.6%
**Kulm**	**AG**	137	1.35	78	43.4%	3	2.2%
**Laufenburg**	**AG**	187	1.19	10	5.2%	10	5.2%
**Lenzburg**	**AG**	113	1.10	34	26.0%	6	5.2%
**Muri**	**AG**	140	1.01	7	4.9%	13	8.9%
**Rheinfelden**	**AG**	90	0.81	6	6.4%	3	3.3%
**Zofingen**	**AG**	204	1.44	62	26.2%	4	1.9%
**Zurzach**	**AG**	105	0.81	3	2.8%	5	4.7%

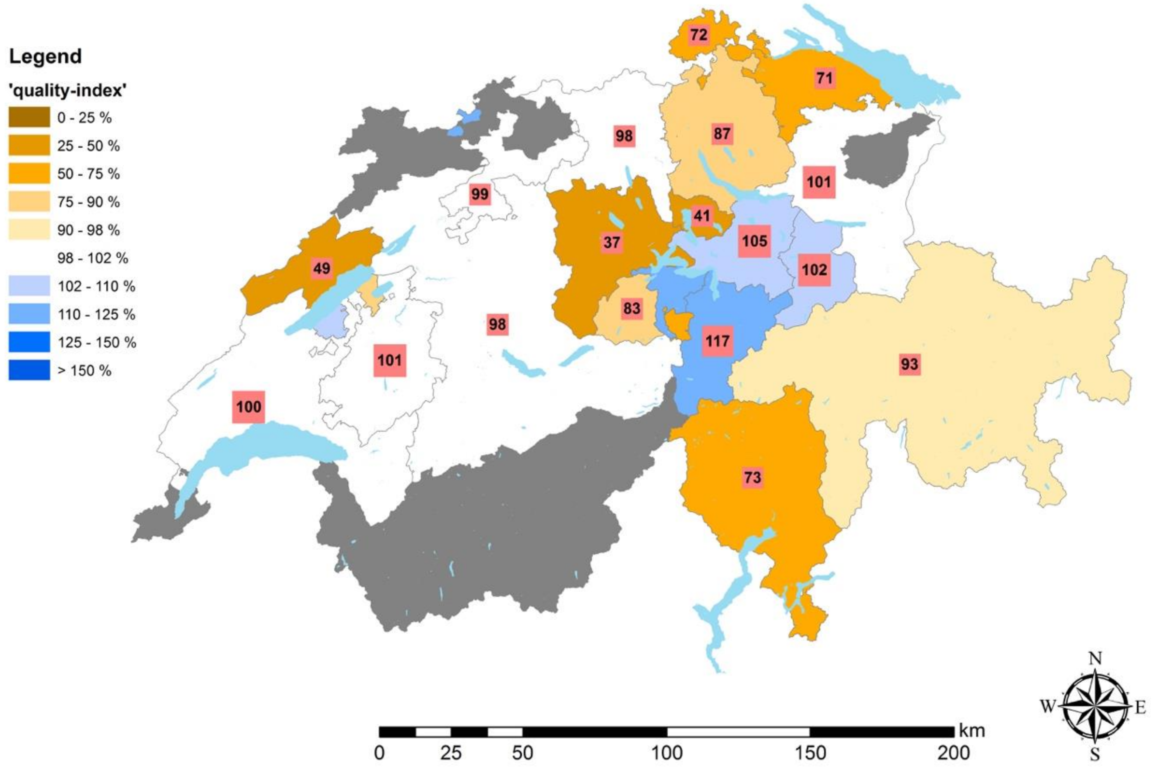

Figure S1: 'Quality-index' on cantonal level. The 'quality-index' gives the proportion of the number of apiaries of the FOAG data set, relative to the number of apiaries of the cantonal data set. Thus, a 'quality-index' of 100% means that there is the same number of apiaries in both data sets. A 'quality-index' > 100% indicates more FOAG-apiaries; < 100% indicates more apiaries in the cantonal data set. Cantons where only one data set was available are indicated in grey (no data to compare).

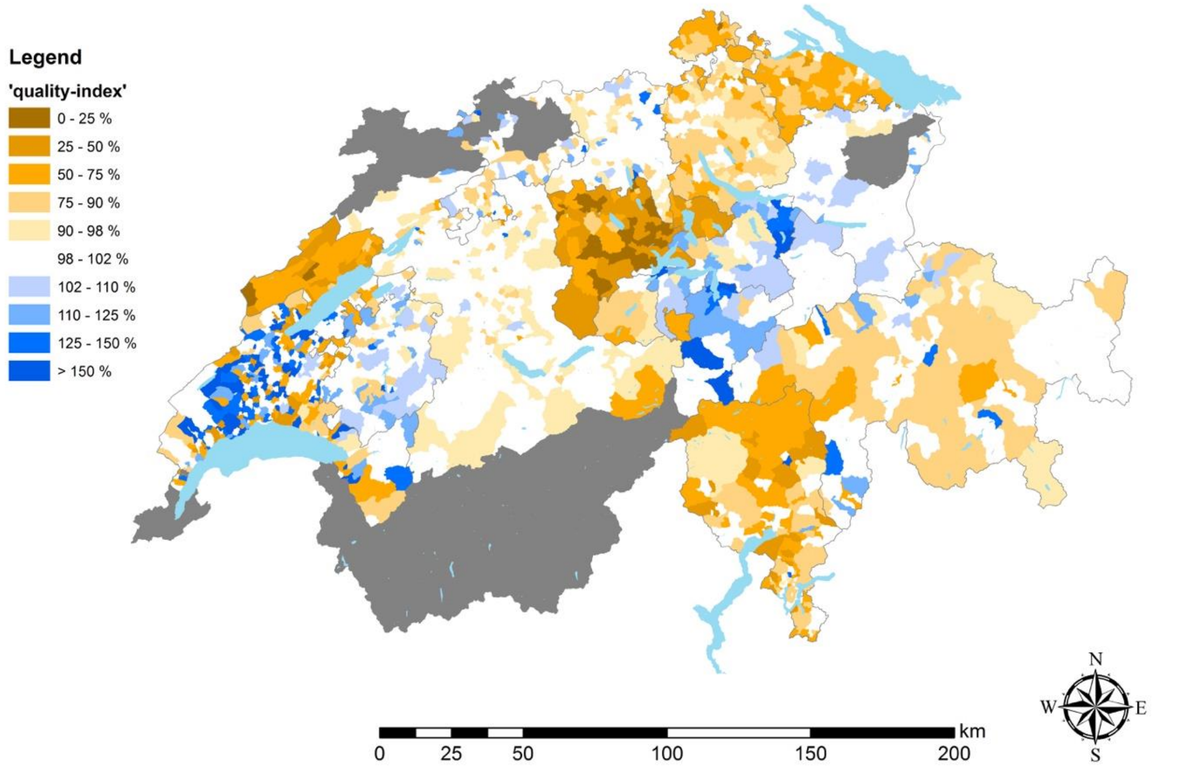

Figure S2: 'Quality-index' on municipal level. The 'quality-index' gives the proportion of the number of apiaries of the FOAG data set, relative to the number of apiaries of the cantonal data set. Thus, a 'quality-index' of 100% means that there is the same number of apiaries in both data sets. A 'quality-index' > 100% indicates more FOAG-apiaries; < 100% indicates more apiaries in the cantonal data set. Municipalities of cantons where only one data set was available are indicated in grey (no data to compare)**.**

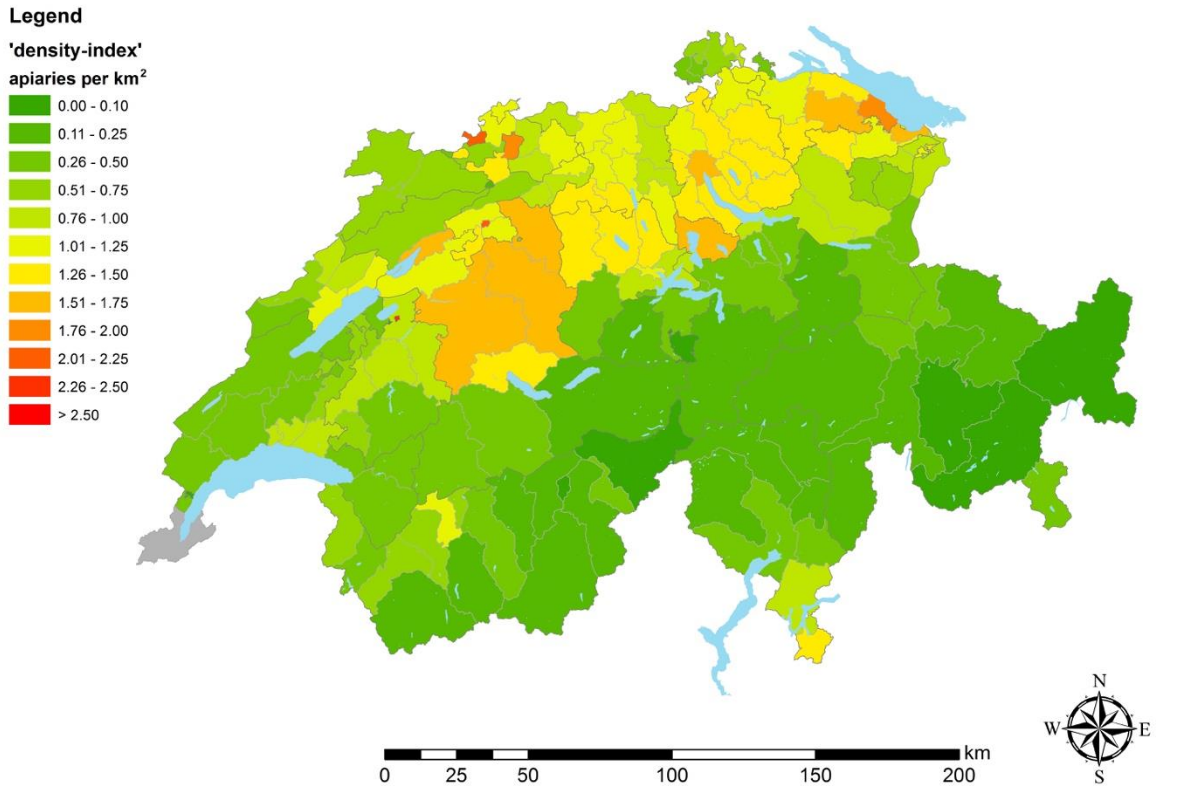

Figure S3: 'Density-index' on district level (2016). Low data quality lead to exclusion of Geneva (indicated in grey).

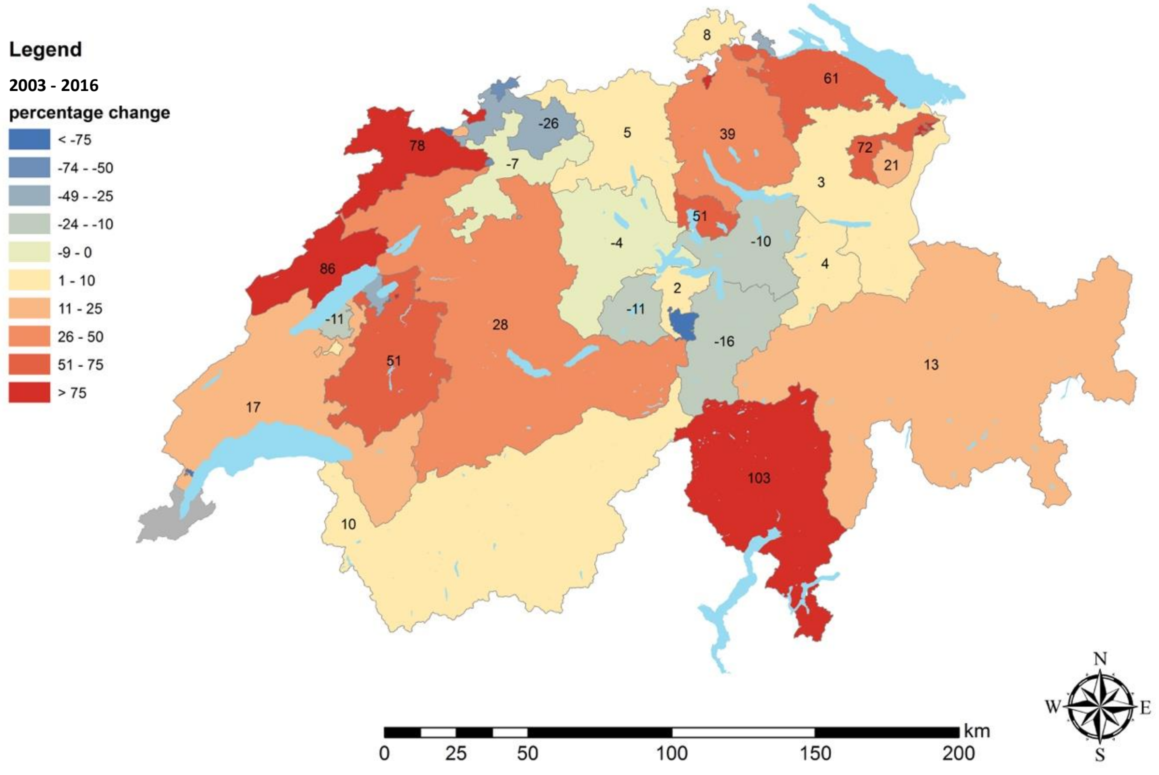

Figure S4: Percentage change for the apiary ‘density-index’ 2003-2016.

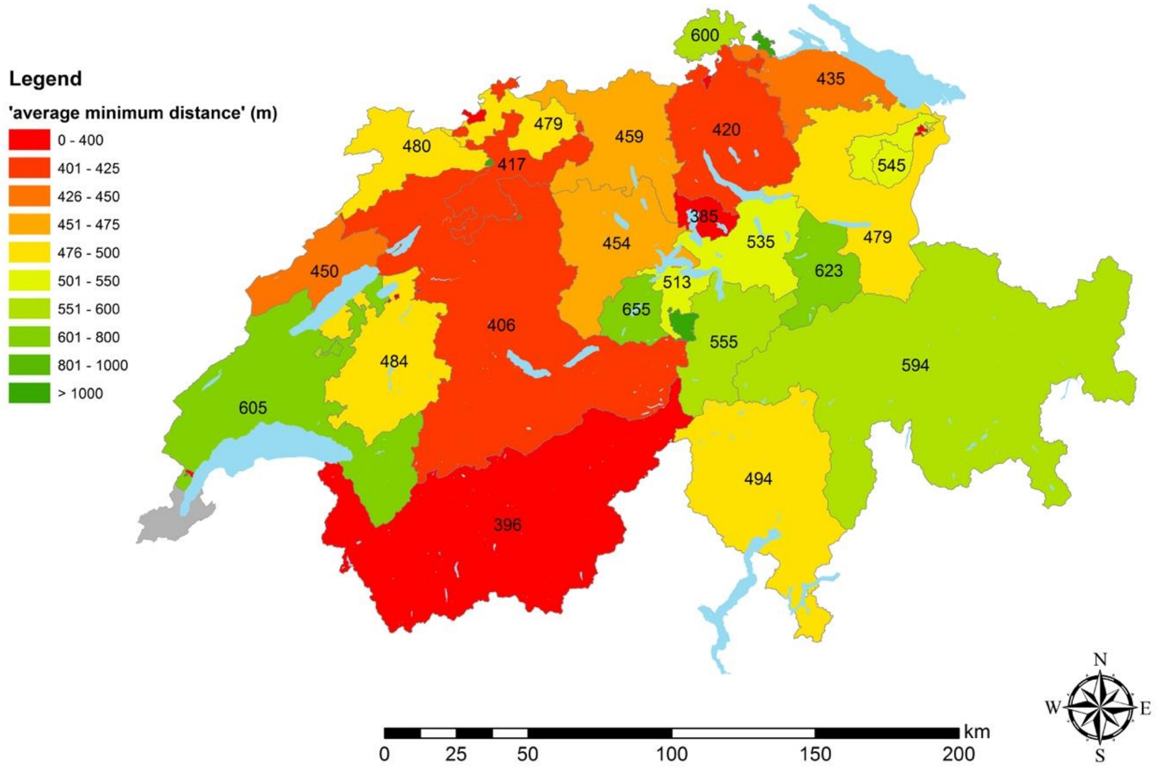

Figure S5: ‘Average minimum distance’ to the nearest apiary on cantonal level.

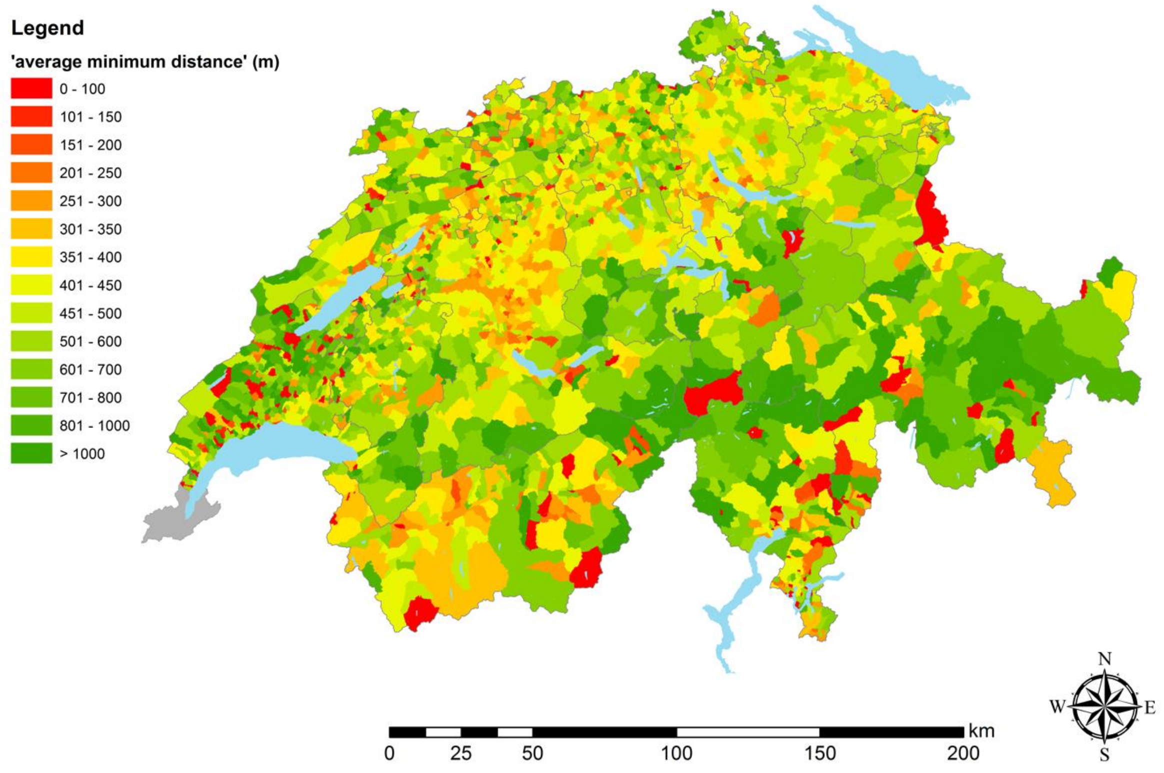

Figure S6: 'Average minimum distance' to the nearest apiary on municipal level.

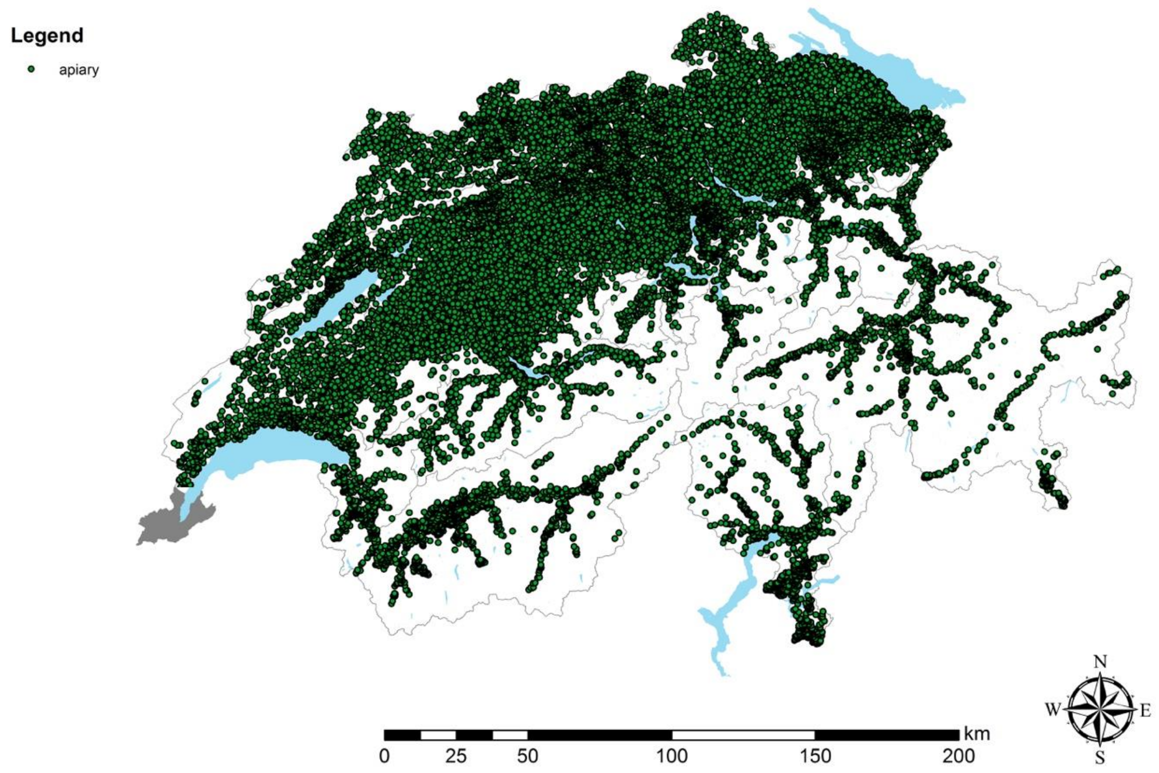

Figure S7: Active apiaries of Switzerland in the end of 2016: 23,055.

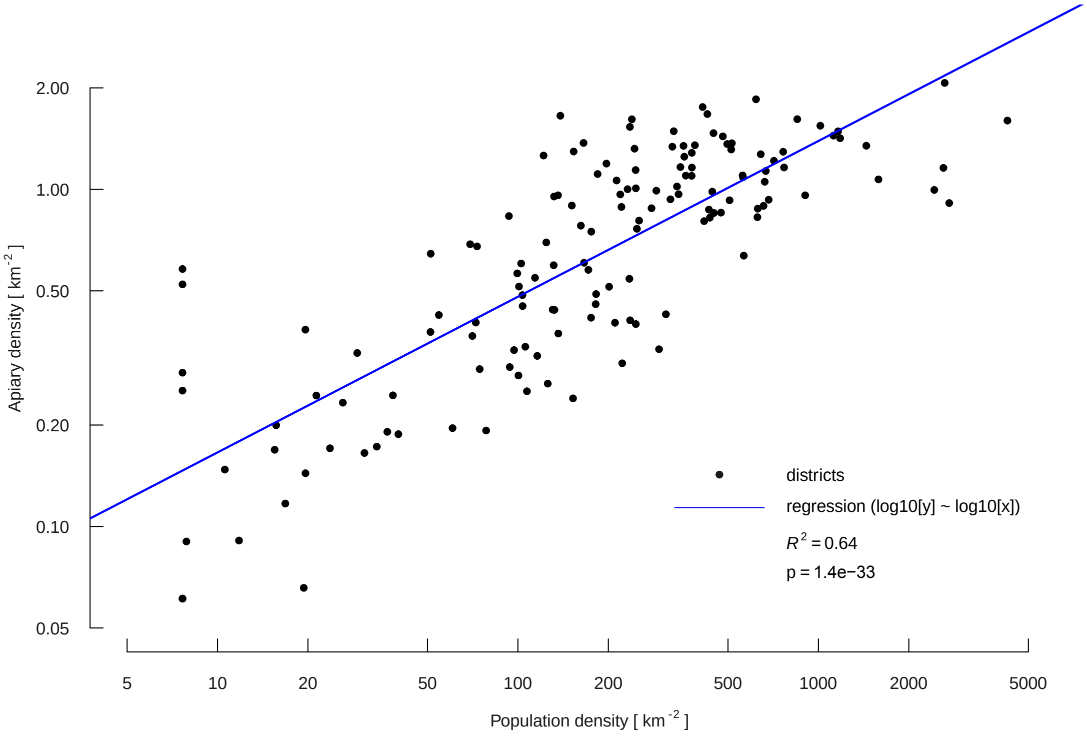

Figure S8: Scatter plot apiary density vs. human population density. Each data point is a district in Switzerland.

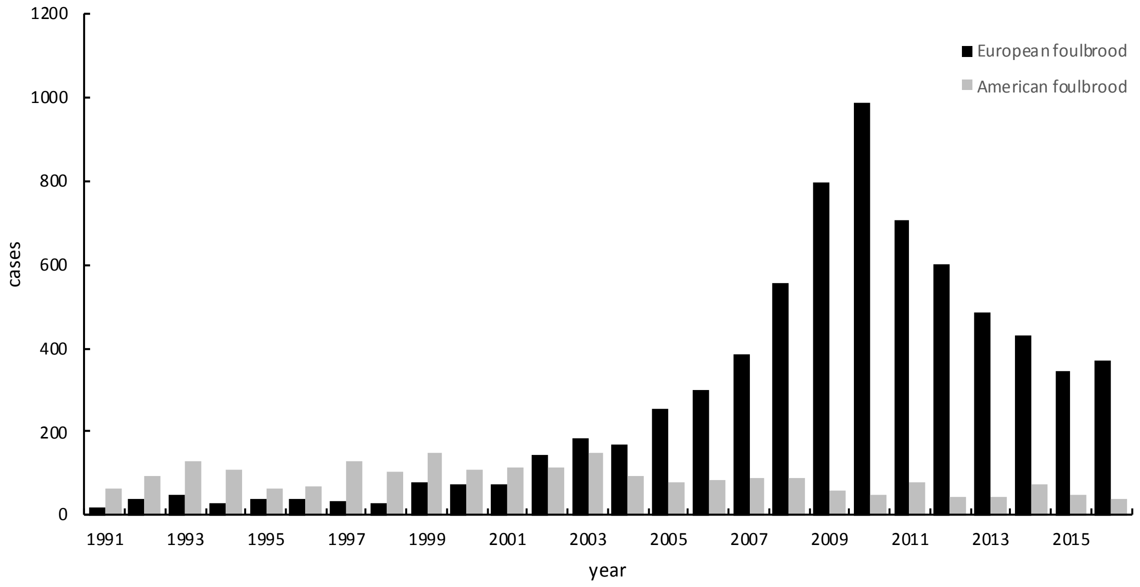

Figure S9: Bar plot of all AFB and EFB cases in Switzerland, summarized for the period 1991-2016.

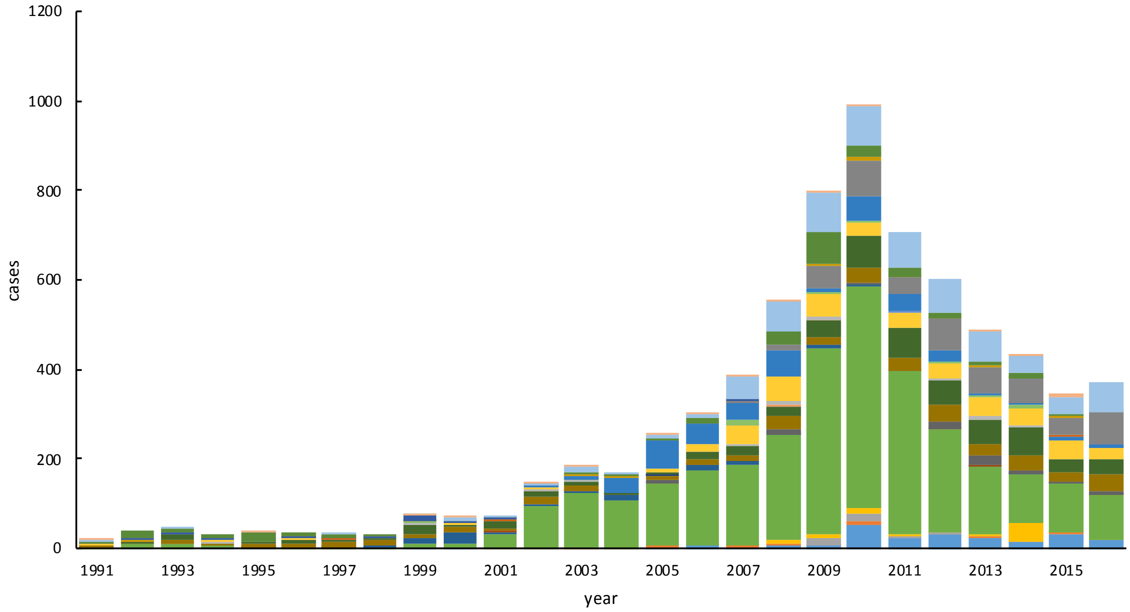

Figure S10: Bar plot of all EFB cases in Switzerland for the period 1991-2016. Different colors indicate different cantons. Light green is Bern, grey is Thurgau and light blue is Zurich.

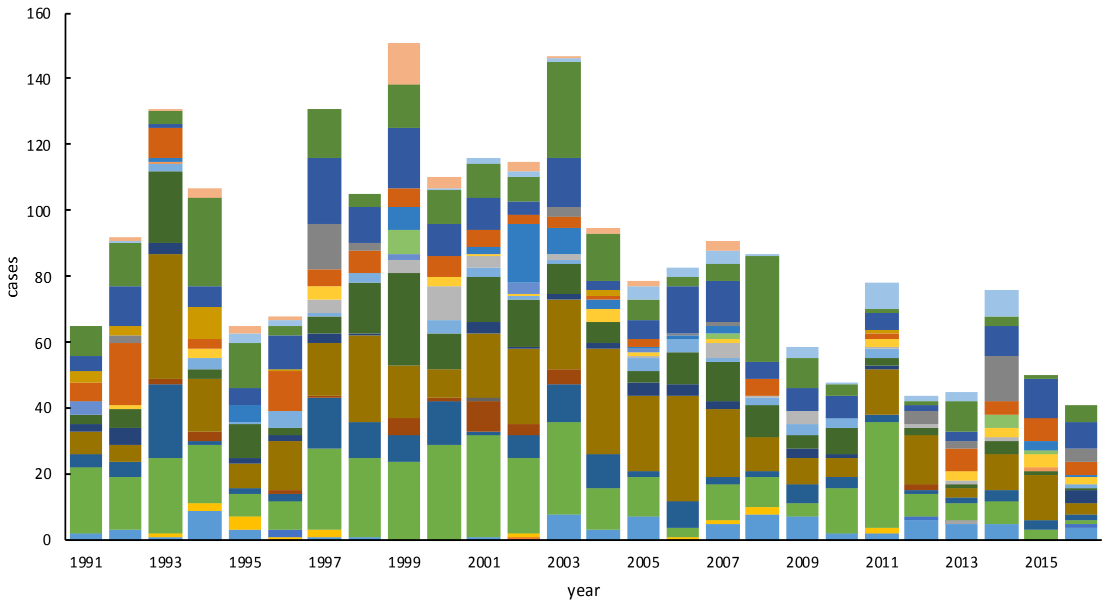

Figure S11: Bar plot of all AFB cases in Switzerland for the period 1991-2016. Different colors indicate different cantons. Light green is Bern, brown is Graubünden, and dark green is Lucerne.
